# Ultrasensitive Optical Fingerprinting of Biorelevant Molecules by Means of SERS-Mapping on Nanostructured Metasurfaces

**DOI:** 10.3390/bios13010046

**Published:** 2022-12-28

**Authors:** Elizaveta Kozhina, Sergey Bedin, Alexander Martynov, Stepan Andreev, Alexey Piryazev, Yuri Grigoriev, Yulia Gorbunova, Andrey Naumov

**Affiliations:** 1 Laboratory of Plasmonics, Skolkovo Institute of Science and Technology, Bolshoy Boulevard 30, bld. 1, 121205 Moscow, Russia; 2 Department of Advanced Photonics and Sensorics, Lebedev Physical Institute RAS, Troitsk Branch, Fizicheskaya Str. 11, 108840 Moscow, Troitsk, Russia; 3 Laboratory of Physics of Advanced Materials and Nanostructures, Moscow State Pedagogical University, Malaya Pirogovskaya St. 1-1, 119991 Moscow, Russia; 4 Laboratory for the Growth of Thin Films and Inorganic Nanostructures Center of Crystallography and Photonics of RAS, Leninskiy Prosp. 59, 119333 Moscow, Russia; 5 Frumkin Institute of Physical Chemistry and Electrochemistry, Russian Academy of Sciences, Leninskiy Prosp., 31 Building 4, 119071 Moscow, Russia; 6 Department of Chemistry, Moscow State University, Leninskie Gory, 1, 119991 Moscow, Russia; 7 Research Center of Genetics and Life Sciences, Research Direction–Biomaterials, Sirius University of Science and Technology, 1 Olympic Ave, 354340 Sochi, Russia; 8 Kurnakov Institute of General and Inorganic Chemistry, Russian Academy of Sciences, Leninskiy Prosp., 31, 119991 Moscow, Russia; 9 Laboratory for Spectroscopy of Electronic Spectra of Molecules, Institute for Spectroscopy RAS, Fizicheskaya Str. 5, 108840 Moscow, Troitsk, Russia

**Keywords:** SERS, nanowires, track-etched membranes, numerical simulations, Raman mapping, biosensing

## Abstract

The most relevant technique for portable (on-chip) sensors is Surface Enhanced Raman Scattering (SERS). This strategy crashes in the case of large (biorelevant) molecules and nano-objects, whose SERS spectra are irreproducible for “homeopathic” concentrations. We suggested solving this problem by SERS-mapping. We analyzed the distributions of SERS parameters for relatively “small” (malachite green (MG)) and “large” (phthalocyanine, H_2_Pc*) molecules. While fluctuations of spectra for “small” MG were negligible, noticeable distribution of spectra was observed for “large” H_2_Pc*. We show that the latter is due to a random arrangement of molecules with respect to “hot spot” areas, which have limited sizes, thus amplifying the lines corresponding to vibrations of different molecule parts. We have developed a method for engineering low-cost SERS substrates optimized for the best enhancement efficiency and a measurement protocol to obtain a reliable Raman spectrum, even for a countable number of large molecules randomly distributed over the substrate.

## 1. Introduction

In recent years, photonics has been rapidly moving towards portable (wearable) devices [1,2] with the ability to sense extremely low concentrations of substances down to the level of single molecules (SM), making SM microscopy and spectroscopy possible on a smartphone [3,4]. It opens fantastic possibilities for point-of-care testing, including PCR, flow cytometry, the primary diagnosis of various diseases [5,6], and even DNA sequencing with compact low-cost devices [7,8]. A similar opportunity exists in numerous adjacent applications such as sports medicine and doping control, food industry, ecology, security, criminalistics, etc. In addition, there is currently great multidisciplinary interest in the synthesis and characterization of organic macromolecules (e.g., molecular machines, Nobel Prize 2016; Mendeleev Prize, 2021) [9,10]. To enable synthesis of macromolecules with desirable properties, one needs to have instrumental methods for quick chemical analysis.

Usually, the methods used are mass-spectrometry and chromatography, which are bulky and relatively insensitive. In contrast, Raman scattering (RS) spectroscopy presents the possibility for the unique recognition of compounds (so called “Raman fingerprinting”), but because of their low intensity it is experimentally difficult to measure them.

To increase RS cross-sections, two approaches are usually used: tip-enhanced RS (TERS) and surface-enhanced RS (SERS). TERS enables even the imaging of vibrations inside a certain molecule [11] or a quantum dot [12,13], whereas SERS can form the basis of the easy-to-use portable platform for extremely sensitive spectrochemical analysis [14,15]. It can be realized by preparing either active colloid nanoparticle solutions [16], or plasmonic substrates (with regular [17] or random [18,19,20,21] metal nanostructure arrays) [22].

The dominant SERS enhancement mechanism (in addition to chemical) is the electromagnetic one (EM) [23], where local electric fields are enhanced in so-called hot spots [24]. The enhancement properties depend on the nanostructures’ morphology [25,26,27,28]. A tip hot spot appears on sharp edges, whereas the gap hot spots are between adjustable nanostructures, where the maximum enhancement is observed [29,30].

A SERS approach with colloidal nanoparticle solutions [31] is very widespread due to the simplicity of their manufacture. However, there are disadvantages in the use of colloidal solutions; they tend to clusterize and show a random orientation and the analyte molecules also lead to the non-stability of recorded SERS signals [32,33].

Much higher efficiency of SERS can be achieved by using nanostructured plasmonic metal surfaces, where both the tip and gap hot spots are possible [34,35]. One such example is nanostructured substrates with an array of nanowires (NW) on the surface [36,37]. With sufficient elasticity, adjacent vertical-standing NWs lean to each other with their tips, where a gap hot spot is formed in the area between the tips [38,39].

SERS overcomes two major drawbacks of Raman spectroscopy: low sensitivity and fluorescence interference, meaning that SERS enhancement can quench the fluorescence background and improve the signal-to-noise ratio [40,41,42]. Thus SERS can be used for a comprehensive understanding of biological processes and early diagnosis of diseases [43], in analytical biochemistry [44,45,46], and even using spectrometer-on-chip concepts [47].

Thus, SERS appears to be the most relevant way to construct an SM chemical sensor. This strategy, however, crashes in the case of large (biomimetic, biorelevant) molecules and nano-objects, whose SERS spectra are irreproducible for extremely low concentrations of analyte [48].

In general, at a high concentration of the testing substance, we get an averaged spectrum from several molecules located in some relation to hot spots of the SERS surface. At a low concentration and countable number of molecules, SM SERS spectra obviously occur with randomly varying intensities and frequencies [49]. Molecules located at hot spot areas would display higher vibrational dephasing rates (and thus smaller oscillation frequencies) [50]. In addition, a Stark shift may occur, owing to the contact potential between electric fields induced by a plasmonic tip and a molecule [51,52].

The situation becomes even more complicated with large macromolecules, because a characteristic variation in the spectra can appear due to the enhancement of different molecular vibrational bonds, which are located near the hot spot. This effect has been demonstrated using the TERS spectroscopy technique, where different SM vibrational bonds in its different areas were imaged [11,53].

Additionally, the detected SERS signal results from the summation of many SM signals located within the probed surface area, both from hot-spot areas (where enhancement is maximal) and the rest of molecules located far from hot spots [29]. One way to decode different contributions to the integral SERS is by the so-called mapping of a SERS substrate [54,55]. In this technique, a series of SERS spectra are measured from all the points of the substrate by sequential scanning using a confocal microscope. This yields many SERS spectra that can improve the limit of detection [56,57,58,59] and take into account all the variants of RS enhancement on the SERS substrate. In spite of the obvious advantage of this technique, its use is not so widespread [60]. There have only been a few studies carried out, and the possibility of sensing large (macro)molecules on the SERS substrate is practically not discussed.

In the present paper, we have shown how the SERS-mapping technique can be used to comprehensively study the spectra of large molecules, as well as calculate the percentage of the active substrate area that enhances the analyte spectra in the case of molecules with a diameter of several angstroms and a nanometer. We suggest an original way to realize chemical sensing and identification of large (biorelevant) molecules by using SERS-mapping on silver-NW metasurfaces, prepared by template synthesis in nanopores of tracking polymer membranes. The method has been tested with relatively “small” (malachite green) and “large” biorelevant (phthalocyanine bearing eight bulky 2,6-diisopropylphenoxy groups, H_2_Pc*) molecules. We consider that one can measure RS-spectra for a large number of molecules randomly disseminated over the SERS substrate and obtain a robust dataset for all possible orientations and locations in relation to hot spots. For a large number of molecules, one can simply statistically enhance RS-spectral peaks for all the possible molecular vibrations, thus obtaining a true RS-spectrum of the analyte by spectrum summation. We anticipate great applicability of the developed approach for various fields, including lab chemistry, pharmaceuticals, and personalized medicine.

## 2. Materials and Methods

### 2.1. Prepare SERS Substrates

Substrates with the Ag-NWs array for SERS spectroscopy were fabricated by template synthesis [61] on a commercial PET (polyethylene terephthalate) track-etched membrane (TM) (produced in Flerov Laboratory of Nuclear Reactions, Joint Institute for Nuclear Research, Dubna, Russia) [62,63] with a surface pore density of 8.4 × 10^9^, 1.2 × 10^9^ and 4 × 10^8^ cm^−2^ for the arrays of Ag-NWs with 60, 100 and 200 nm in diameter, respectively. For synthesis details, see Supplementary Materials.

The morphology of samples was studied using a dual-beam field emission scanning electron–ion microscope Scios (0.5–30 kV) (FEI, Hillsboro, OR, USA). The NW length, diameter and features of bundle formation were determined.

**Figure 1:**
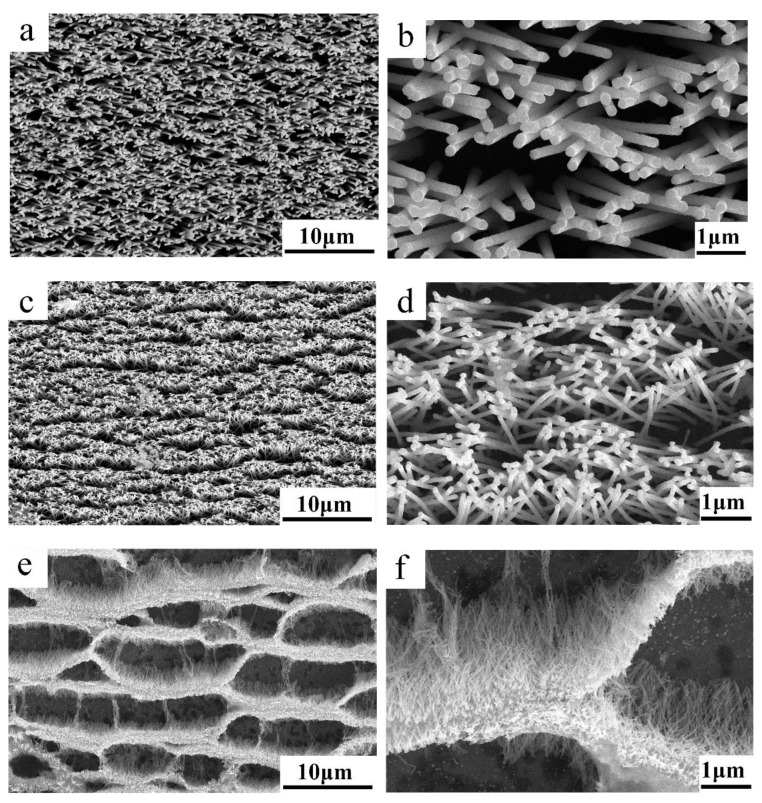
SEM images of the substrate surface with NWs of 200 (**a**,**b**), 100 (**c**,**d**), and 60 nm (**e**,**f**) diameter and 10 µm length.

Figure 1 shows SEM images of metasurfaces with vertically standing NWs with different diameters and surface pore densities. On the substrate with the largest chosen NWs diameter (200 nm), NWs almost exactly reproduce the pore structure of the original TM (Figure 1a). As the diameter decreases to 100 nm (Figure 1c), NWs become more elastic and tend to agglomerate [64,65]. At the same time, due to the peculiarities of the TM production, agglomeration occurs in the form of characteristic strips. On substrates with the smallest chosen NW diameter of 60 nm (Figure 1e), the NWs are highly flexible, and, upon drying, the substrates tend to form agglomerates in the form of a cellular structure.

Due to the high elasticity of NWs with a diameter of 60 nm and a length of 10 µm, under the influence of capillary forces, the formation of strips consisting of leaning adjacent NWs by their tips occurs (Figure 1e). As a result, the adjacent NWs are closely connected and form a cellular structure. For NWs with a diameter of 100 nm, hot spots are formed as strips that do not intersect each other (Figure 1c). The number of NWs that form a strip on these substrates is less compared to an array of 60 nm NWs, which makes it possible to generate high-intensity local electric fields in the gaps between the nanowire tips.

NWs with a diameter of 200 nm are not elastic, and the magnitude of capillary forces is not enough for the tips of adjacent NWs to be close to each other (Figure 1a). As a result, the pattern of distribution of hot spots is not a strip consisting of leaning NWs, but autonomous bundles, including units of NWs (Figure 1b). The probability that a molecule is adsorbed in the hot-spot region depends mainly on the effective adhesion area of the NWs, which means that the process of forming a pattern of hot-spot distribution on a SERS-active metasurface is essential.

Since hot spots are formed in the contact area of the NW tips, we can conclude that the largest one is in the case of 60 nm NWs (Figure 1). However, due to the high density of 60 nm NWs, several hundreds of NWs form a bundle, which prevents the formation of well-defined contact point between the NW tips. Therefore, no high-intensity local electric fields appear in the contact region. Thus, to create a working SERS-active metasurface with an array of 60 nm NWs, it is necessary to synthesize them with much shorter lengths.

From the results of electron microscopy, we determined that, depending on the NW diameter and surface pore density, it is possible to obtain metasurfaces with different levels of agglomeration.

### 2.2. Molecules and Samples

From the chemical viewpoint, the range of objects for SERS studies is almost unlimited, varying from the simplest aromatic molecules, like thiophenol, to huge biomolecules and even viruses [66]. However, fundamental studies of the SERS phenomenon are often limited to relatively small yet industrially and biologically important model molecules, such as dyes, antibiotics, pesticides, etc. [67,68].

Diversification of molecular sizes of model compounds can be used to reveal the dimensional effects associated with the interaction between SERS substrates and analytes. In this context, in the present work, we used two dyes with significantly different molecular dimensions—malachite green (MG; chemical formula C_23_H_25_ClN_2_), and a phthalocyanine bearing eight bulky 2,6-diisopropylphenoxy groups (H_2_Pc*; chemical formula C_128_H_146_N_8_O_8_).

MG is an organic dye with a well-known spectrum, allowing comparison of the results objectively with other works [69,70]. The solvent chosen was distilled water. The MG concentration was 27 µM and the drop volume was 3 µL. The required amount of H_2_Pc* (0.1 mM) was dissolved in distilled chloroform.

**Figure 2:**
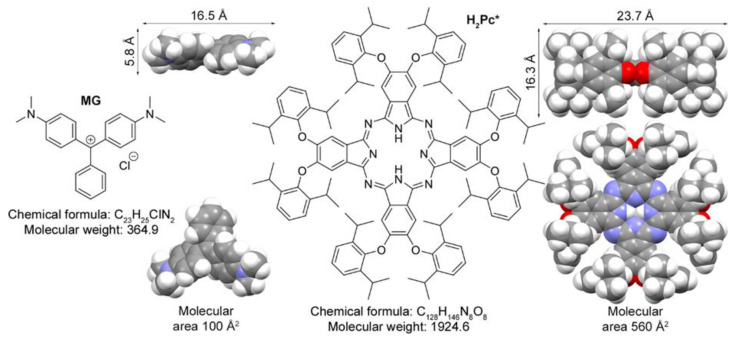
Compounds studied in the present work–malachite green dye, MG; and octa (2,6-diisopropylphenoxy) phthalocyanine, H_2_Pc*. Molecular sizes are based on space-filling models obtained by geometrical optimization at the BP86/def2-SVP level of theory (only triphenylmethyl cation was optimized in the case of MG).

Quantum-chemical modelling suggests that the molecular area of H_2_Pc* is 5.6 times larger in comparison with the MG cation (Figure 2), and we will show that this difference is sufficient to detect the difference in the generation of a SERS signal. While MG is a typical object for SERS studies [71] because of its ecological relevance, the choice of the phthalocyanine is dictated by the huge impact of this class of compounds on materials and life sciences, including photodynamic therapy and diagnostics, photovoltaic technologies, sensing applications, etc. [72]. For the synthesis and computational details, see Supplementary Materials.

### 2.3. SERS Spectroscopy and Mapping

The experiments were carried out on a Horiba LabRam Evolution Raman spectrometer equipped with a single-mode CW-laser for Raman spectrum excitation in the confocal microscope scheme with a spatial amplification of ×100. The working laser wavelength was 532 nm, with a maximum power of 170 mW. In the measurements, we used 1% of the maximum possible laser power (1.7 mW).

The control experiment for the case of phthalocyanine molecules was performed on another Raman spectrometer, a Renishaw inVia™ Confocal Raman microscope, at an excitation wavelength of 1064 nm (see Supplementary Materials). In that wave range, phthalocyanine molecules have a low photoluminescence contribution. The data for each spectrum were acquired by the sequence of three measurements with an exposure time of 60 s for each one. In the measurements, we used 1% of the maximum possible laser power for such equipment.

Selection of the proper laser wavelength is quite important in order to diminish the contribution of photoluminescence of the analyte which can interfere with the detection of Raman spectra; this is important especially in the presence of plasmon nanostructures, which can quench PL, as well as enhance it due to the Purcell effect [73].

The data for each spectrum were acquired by the sequence of three measurements with an exposure time of 5 s for each one. Spectra were processed using Spectragryph software [74].

SERS-mapping was realized by standard XY-scanning in the confocal scheme of the used Raman spectrometer in the SWIFT mode with an exposure of 0.5 s for each point. Scanning range was of 20 × 20 µm, and a total of 400 SERS spectra were taken in that area with steps of 1 µm in both directions (which roughly corresponded to the diffraction limited volume). The mapping technique can be fully (or semi-) automated. Hundreds of spectra were measured and collected at every point (pixel) of the defined area, and then used to generate artificial color images based on the intensity of a designated Raman peak.

## 3. Results and Discussion

### 3.1. Physical Mechanisms of the SERS Enhancement Effect

Numerical calculations of the enhancement of the local electromagnetic fields near the gap between two Ag-NW tips were performed using the electromagnetic KARAT code in planar geometry (X, Z) with a monochromatic incident laser wavelength of 532 nm [75]. The Drude model was used, taking parameters for silver: a plasma frequency of 8.78 eV and a damping constant of 0.02 eV.

**Figure 3:**
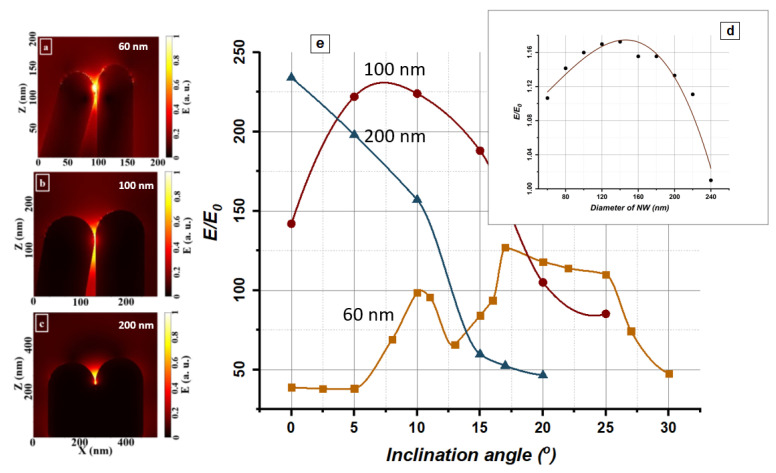
(**a**–**c**) Electric field enhancement (E/E0) in the area between leaning NW tips: (**a**) nanowire diameter of 60 nm, tilt angle of 20 degrees; (**b**) nanowire diameter of 100 nm, tilt angle of 10 degrees; (**c**) nanowire diameter of 200 nm, tilt angle of 10 degrees; (**d**) The dependence of the electric field enhancement on the nanowire diameter (excitation is under the 532 nm CW laser); (**e**) Dependence of the maximum local field enhancement (E/E0) in the area between leaning NW tips on the tilt angle.

To determine the optimal geometric parameters of NWs, the local electromagnetic fields near a single nanowire were calculated. Figure 3d shows the dependence of the electric field-enhancement factor (E/E0) near a single Ag-NW at its diameter. As can be seen from the plot, the maximum enhancement is approximately equal to 1.2 and corresponds to a NW diameter of about 120–140 nm. The local enhancement of electric fields turns out to be orders of magnitude greater for hot spots formed in nanometer gaps between leaning NWs.

For NW diameters of 60, 100, and 200 nm, the enhancement of local electromagnetic fields in the area between two leaning NW tips was simulated. Linearly polarized laser radiation (Ex, By) with a wavelength of 532 nm was propagated in the positive direction of the Z-axis. One of the nanowires was located along the normal to the incident radiation, and the other was located at a tilt angle to the first one, forming a gap with a width of 1 nm. Figure 3a–c shows the electric field-amplitude distribution, normalized to the amplitude of the incident radiation E0. The NW lengths differ by 2 nm, reflecting that, under actual experimental conditions of the NW template synthesis, their lengths slightly vary. As seen from the modeling, the maximum enhancement of the electric field amplitude occurs in the gap between neighboring NW tips. It is interesting to study how the maximum field enhancement factor (E/E0) in the gap depends on the NW tilt angle. According to SEM images (Figure 1), the tilt angles for NWs with diameters of 60 and 100 nm vary over a wide range; from 0 to 30° or more. In comparison, NWs with a diameter of 200 nm cannot form contacts at angles greater than 20° due to their limited elastic properties.

Figure 3e shows the dependence of the maximum local field enhancement (E/E0) in the gap between NW tips on the tilt angle for NWs with diameters of 60, 100, and 200 nm. It can be seen from the plot that for NWs with a diameter of 60 and 100 nm, a significant local field enhancement factor is observed in a relatively wide range of tilt angles. E/E0 reached a maximum of 127 at a tilt angle of 17° for 60 nm NWs, and 224 at a tilt angle of 10° for 100 nm NWs. For NWs with a diameter of 200 nm, the maximum enhancement factor equal to 234 is achieved at a tilt angle of 0°. As shown in [66], the SERS signal enhancement factor (EF) relates to the field enhancement as (E/E0)^4^, considering its average over all illuminated molecules. Thus, the maximum EF for leaning NWs is approximately 10^8^–10^9^.

The dependence of the field enhancement on the NW diameter at a fixed tilt angle has a rather complex form. At small angles (less than 5°), the highest enhancement is observed for NWs with the largest diameter. In this case, the rounded ends of NWs play the role of nanoantennas—electromagnetic field concentrators: the larger their area, the greater the field enhancement of a localized surface plasmon in the space between the NW tips (gap hot spot).

**Figure 4:**
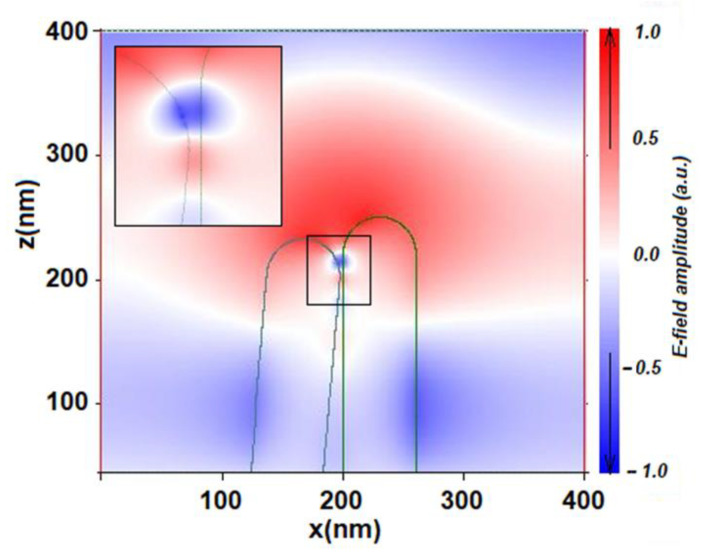
Formation of a propagating plasmon–polariton (alternating blue and red areas) near the space between two NW tips with a tilt angle of 5°.

For NWs with diameters of 60 nm and 100 nm at tilt angles greater than 5°, in addition to the gap hot spot, conditions for the formation of a propagating surface plasmon-polariton (SPP) arise (Figure 4). In this case, NWs play the role of plasmonic waveguides, along which SPP can propagate over considerable distances [76]. This effect leads to an additional enhancement of the electric field amplitude and the appearance of maxima in the corresponding curves in Figure 3e in the range of tilt angles from 5 to 25°. Note that SPP does not arise for 200 nm NWs. Therefore, the field enhancement factor, in this case, decreases monotonically with increasing tilt angle.

The performed calculation shows that NWs with a diameter of 60 and 100 nm are optimal for amplifying the SERS signal since they can provide a sufficiently large enhancement over the entire substrate area due to their dependence on the tilt angle. At the same time, NWs with a diameter of 200 nm will provide SERS amplification signal only in those areas of the substrate where NWs are leaning towards each other at minimal angles close to zero.

Note that, in addition to the NW diameter and tilt angle, the size of the field localization region is also an important parameter that determines the number of molecules trapped in the enhanced field region. On the one hand, the larger the gap between NWs (gap width), the larger the area of the enhanced field region. On the other hand, the smaller the gap width, the higher the electric field-enhancement factor. In our previous work, we have calculated that the optimal value of the gap width is in the range of 10 to 15 nm, providing both a sufficiently large electric field enhancement and a significant number of substance molecules trapped in the region of effective amplification. This is especially important for large molecules such as phthalocyanine, whose size coincides in order of magnitude with the gap between nanowires.

### 3.2. SERS-Mapping with Small Molecules

Let us consider the measured Raman spectra of the standard organic dye MG, both on glass and SERS substrates.

**Figure 5:**
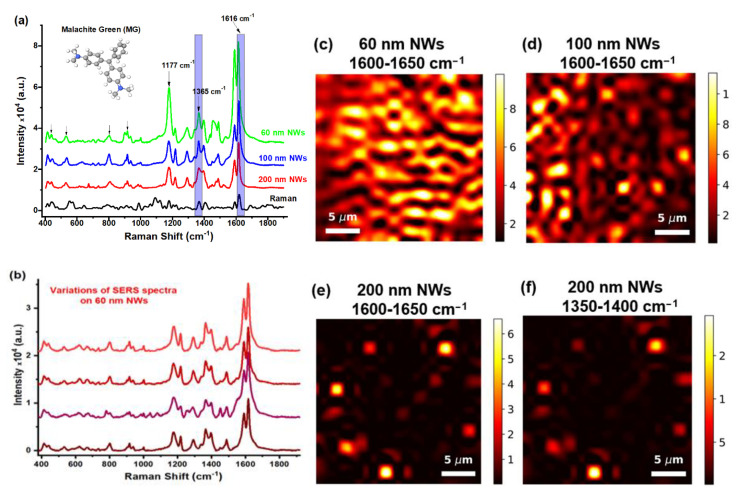
(**a**) Raman spectrum of 0.27 mM MG solution multiplied by 10, then SERS spectra of 0.27 µM MG solution on substrates with NWs with a diameter 200 nm, 100 nm and 60 nm. (**b**) SERS spectra recorded from different parts of the substrate with 60 nm diameter NWs. (**c**–**e**) SERS-mapping of substrates with NWs with a diameter of 60, 100, and 200 nm, which was plotted from the area under the peak located at 1616 cm^−1^. (**f**) SERS-mapping of substrates with an NW of diameter 200 nm, which was plotted from the area under the peak located at 1365 cm^−1^.

SERS spectra of MG molecules adsorbed on substrates with an array of NWs with a diameter of 60, 10, and 200 nm are shown in Figure 5a. The strongest SERS peaks were found at 1589, 1616 and 1177 cm^−1^. The first two are assigned to combinations of the C=C stretching vibrations of the phenyl ring, and the first one is the C-H bending mode of the phenyl ring. The other peaks at 434, 530, 802, 916, 1290, 1363 and 1487 (combination of the C=C stretching motions of the aromatic ring), are also present in the spectrum, which agrees with other experimental and theoretical studies [69,77,78,79]. Comparison of the obtained SERS spectra of the MG solution at a concentration of 27 µM with the Raman spectrum of MG at a higher concentration of 0.27 mM (Figure 3a) shows that the SERS signal intensity increases with a decrease in the NW diameter. The most efficient substrates are those with NWs 60 nm in diameter (Figure 5a). This can be explained by the feature of the hot-spot formation by NWs of different diameters. Due to the small size of MG molecules, the size of the enhancing area of the substrate will play the main role in getting the most intense SERS spectra.

Figure 5c–e shows the SERS-mapping of 60, 100, and 200 nm-diameter NW substrates, which was plotted by the area under the peak located at 1616 cm^−1^, since this is one of the strongest peaks. It is clearly seen that substrates with an NW diameter of 60 nm are the most efficient due to the formation of NW agglomerates in the form of a cellular structure. At the same time, NWs with a diameter of 200 nm are the least effective since there are very few areas where such NWs lean together, in addition to hot spots. 

It should be especially noted that there is no variation in the position of SERS peaks, which is demonstrated by a set of spectra of MG adsorbed on NWs with a diameter of 60 nm at different amplifying parts of the substrate (Figure 5b). This directly indicates the stability of the SERS signal amplification and characterizes the enhancing stability of the substrate. This is likely due to the feature of the technique for fabricating SERS-active substrates by the template synthesis method, which provides a high level of reproducibility of the substrate structure over a large area.

The set of vibrational modes of the MG dye is small, and if the molecule hits the amplifying surface, then all peaks will be uniformly enhanced. This is demonstrated by the SERS-mapping of MG molecules on the substrate with 200 nm diameter NWs (Figure 1e,f). The enhancing regions are located in the same parts for three different characteristic MG peaks.

Summing up the case of relatively “small” organic molecules (exemplified by MG), it can be stated that the SERS spectra obtained from different parts of the SERS substrate are qualitatively the same, but fluctuations of intensity (i.e., enhancement coefficient) can be strong. The level of fluctuations depends on the nanostructure of the SERS surface and decreases with decreasing diameter of NWs. The most homogeneous map was observed for 60 nm NWs but, even in this case, obtaining a robust, reproducible and recognizable Raman fingerprint requires the mapping of the SERS substrate to find obvious hot spots. It is especially important for single-molecule concentrations of the analyte.

### 3.3. SERS-Mapping with “Large” Molecules

**Figure 6:**
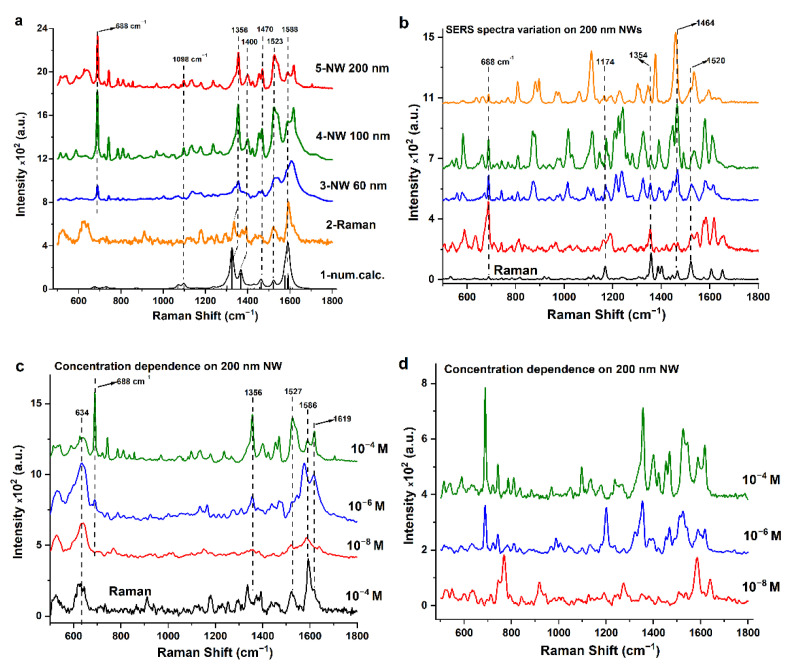
(**a**) Raman spectra of H_2_Pc* (concentration 0.1 mM) as found in quantum-chemical calculations measured on glass substrates, and measured on SERS substrates with different NW diameters (60 nm, 100 nm, 200 nm) obtained by averaging the spectra from all points of SERS substrates. (**b**) Variations of SERS spectra of H_2_Pc* obtained from different points of the SERS substrate with 200 nm NWs. (**c**) Raman spectra of H_2_Pc* with different concentrations as obtained by averaging the spectra from all points of SERS substrates (NW 200 nm) in comparison with an ordinary Raman spectrum from a bulky sample on a glass substrate. (**d**) Raman spectra of H_2_Pc* with different concentrations (NW 200 nm).

As compared with the case of MG, the situation starts to be much more complicated with the large biorelevant substance H_2_Pc*. In Figure 6a, the Raman spectra are shown which are found in quantum chemical calculations, measured from large volumes of the sample on glass substrates, and measured for quite low concentrations of the analyte (0.1 mM) in some random places of the SERS substrates with different NW diameters.

First, we found good agreement between Raman spectra and those obtained by quantum-chemical calculations, which confirms the adequacy of both the calculation and the measurement procedure. The characteristic peaks of H_2_Pc* molecules are present in the entire set of both spectra 1 in Figure 6a. For example, all the peaks are observed at 688, 1174, 1354, 1464 and 1520 cm^−1^. All of these peaks, except the peak at 688 cm^−1^, are the peaks of H_2_Pc* molecules and are responsible for bond vibrations, which are present in large quantities in the composition of the H_2_Pc* structural formula. Calculations suggest that the most intense bands in the Raman spectrum of H_2_Pc* should correspond to vibrations associated with the rigid inner-core polyene with annealed benzene rings (see Supplemental materials for more details). Peripheral substituents have certain conformational freedom associated with rotation around C-C and C-O bonds, which can contribute to inhomogeneity of spectra measured at different points of the substrate, thus emphasizing the need in averaging the spectra over a large number of measurements. The appearance of the peak at 688 cm^−1^ we attribute to the presence of a large fraction of solvent molecules (chloroform), associated with the appearance of agglomerates of H_2_Pc* molecules.

Figure 6b shows SERS spectra obtained from different spatial points of the SERS substrates with 200 nm NWs. One can see obvious quantitative (intensity of peaks) and qualitative (presence/absence of different peaks) differences. We attribute this observation to the size of macromolecules in comparison with hot spots. The signals significantly vary between hot spots with slightly different junction dimensions and orientations. The latter, on the contrary, has its origin in external perturbations contributing to (small) changes in the frequency and width of the peaks of individual molecules (forming part of an overall total measured spectra). These small changes are typically attributed to the diversity of similar (but not identical) conditions that molecules can experience in their interactions with a given environment (hot spot area).

The molecules are adsorbed randomly on strongly inhomogeneous SERS surfaces, and the peaks of those bonds with which the molecule is placed relative to the hot spot will be predominantly observed in the recorded SERS spectrum. This will also lead to difficulty in resolving peaks of individual molecules due to the inhomogeneous distribution of parameters. In addition, multiple molecular events can appear as “frequency shifts” simply because the resolution is not high enough to distinguish the situation from the actual frequency shift of a single molecule. Thus, we find that the frequencies of vibrational modes correlate with the position of the molecule relative to the enhancing SERS-active surface. Also, the frequency shift of the spectral line can occur due to the Stark effect as a result of the difference in the field potential near the local hot spot located near leaning NW tips.

Thus, to obtain robust reproducible Raman spectra, it is necessary to average all the spectra from all points of the SERS map; then the main Raman peaks will be informative. Such a procedure has been performed for SERS substrates with different NW diameters. These averaged spectra are shown on Figure 6a. One can see that these averaged spectra are almost identical to the ordinary Raman and calculated spectra, in contrast to somewhat “stochastic” spectra from different points of the SERS substrate (Figure 6b).

The above-mentioned considerations give the chance to develop correct a procedure for SERS-sensing and identification (fingerprinting) of large biorelevant macromolecules even with an extremely low concentration. The measuring protocol includes SERS-mapping over a large enough square of substrate with subsequent averaging of the spectra. Figure 6c shows the averaged SERS spectra of a solution of H_2_Pc* of different concentrations adsorbed on substrates with 200 nm diameter NWs. Figure 6d shows the random SERS spectra of a solution of H_2_Pc* of different concentrations adsorbed on substrates with 200 nm diameter NWs. Even though substrate parameters are not optimal because of the bad elasticity of 200 nm NWs, and they do not form agglomerates, even a solution of H_2_Pc* with a concentration of up to 10 nM can be detected. It is also worth noting that the solvent peak at 688 cm^−1^, which indicates the appearance of molecule agglomerates, already ceases to be noticeable for a SERS spectrum of 1 µM molecules, and is completely absent in the spectrum of molecules at a concentration of 10 nM. The absence of molecule agglomerates indicates the potential possibility of recording single-molecule SERS spectra.

**Figure 7:**
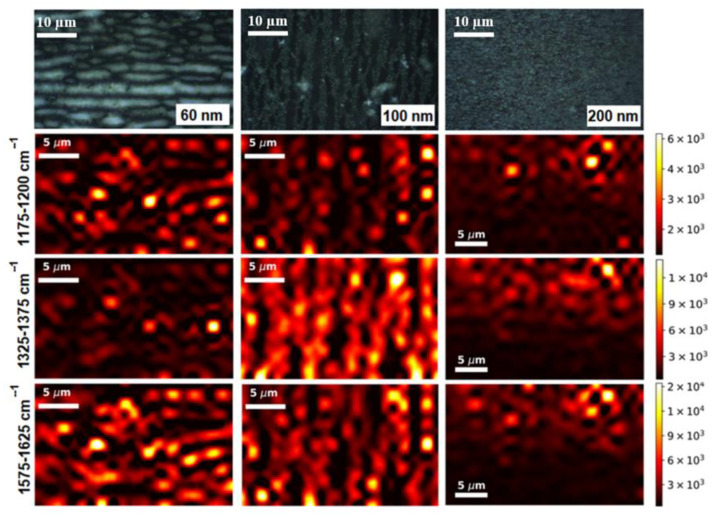
Optical microscopy images of SERS substrates with different NW diameters of 60, 100 and 200 nm (top line of images). SERS-mapping of H_2_Pc* (10 mM) adsorbed on substrates with NWs of 60, 100 and 200 nm in diameter, plotted in accordance with integral intensity (the area) under three characteristic Raman peaks (1175, 1350 and 1600 cm^−1^).

To show how the enhancement efficiency is distributed over SERS substrates with different diameters of NWs, we plotted maps for different specific spectral peaks at 1175, 1350 and 1600 cm^−1^ (see Figure 6). While spectra are strongly fluctuating, for mapping we take the area under the characteristic SERS peak, rather than its intensity, since there is a variation in the position of the peak maximum in the spectra, and we can accidentally get into the noise part of the spectrum. For illustration, the top images in Figure 7 show optical photographs of the substrate surface, on which the H_2_Pc* spectra were recorded to construct the SERS mapping.

It is obvious that, in contrast to small MG molecules, patterns for different Raman peaks are different. This confirms our preliminary consideration that large molecules have a lot of possibilities to be located (oriented) in relation to hot spots. As a result, the SERS peaks of that part of the molecule that is closer to the region with a high-intensity local electric field are enhanced.

**Figure 8:**
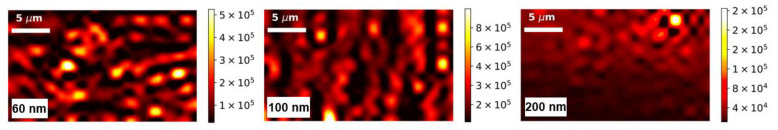
Raman spectral maps of the H_2_Pc* molecules on SERS substrates with different NW diameters plotted over the full area under the spectra from 500 to 1800 cm^−1^.

Figure 8 shows the Raman spectral maps of H_2_Pc* molecules on SERS substrates with different NW diameters plotted over the full area under the spectra from 500 to 1800 cm^−1^. It can be clearly seen that the structure of the area distribution perfectly fits the mapping over integral intensity (the area) under characteristic Raman peaks. This proves the theory about the existence of hot spots in the area between NW tips, because these SERS maps prove that the greatest amplification of the SERS spectrum and the associated concentration of electrical fields is on the NW. Since the focal point in SERS spectroscopy is larger than the size of the hot spot, we cannot say that there are point gaps between the NW tips, but we may say that the presence of hot spots in the place of leaning NW is indisputable. For large molecules, unlike small ones, their orientation relative to hot spots (angular dependencies) is extremely important. In this case, an important role is played by the angles at which neighboring NWs stick together.

**Figure 9:**
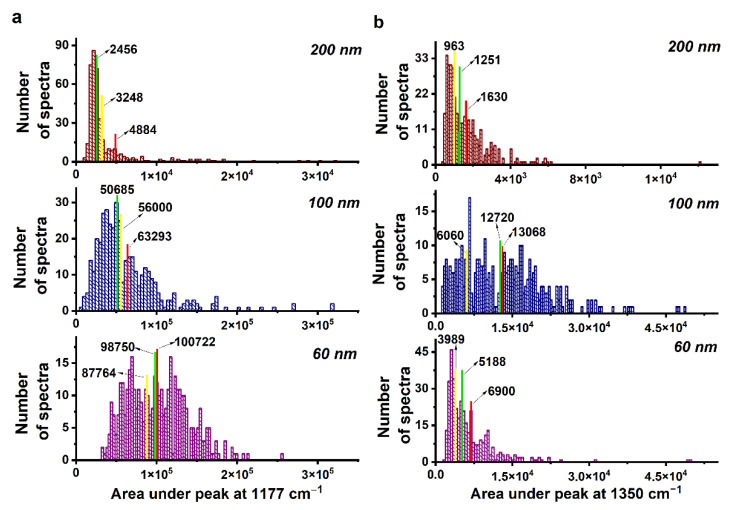
(**a**) Histograms of the distribution of the area value of the MG SERS peak at 1177 cm^−1^. (**b**) Histograms of the distribution of the area value of the phthalocyanine SERS peak at 1350 cm^−1^.

The quality of SERS-active surfaces can also be assessed using characteristic histograms. Figure 9a shows the distribution histograms of the area value of the MG SERS peak located at 1177 cm^−1^. The green line is the histogram median, and the red line is the mean value, where the median is the middle of the set of numbers and the mean value is the average of a data set. With a decrease in the NW diameter, the median of the histogram shifts to the region of large area values which indicates that, for a larger number of sample spectra (400 spectra in total), the area under the peak takes on higher values compared to the spectra of MG molecules adsorbed at NWs 200 nm in diameter. In addition, the relative shift of the mean value relative to the median of the diagram is nearly 50%, 20%, and 2% for NWs 200 nm, 100 nm, and 60 nm in diameter, respectively (see Table 1).

$$\eta =\frac{\mathrm{mean}-\mathrm{median}}{\mathrm{mean}}\times 100$$

To determine the effective enhancing-area value, we need to find the boundary between the spectral noise and amplified SERS spectrum. The distribution histogram shows that there are several characteristic enhancement zones (peaks) for substrates with 60 and 100 nm diameter NWs, as well as one peak for NWs with a diameter of 200 nm. This suggests that the substrates of NWs 200 nm in diameter are the least efficient and most of the SERS spectra are below the noise threshold. For substrates with 60 and 100 nm NWs, the first peak indicates exactly the position of the spectral noise. If we find the half-width of this peak, then this will be the boundary between spectral noise and enhanced SERS spectra. According to the results of the assessment of the effective enhancing-area value of the substrates with NWs 60, 100 and 200 nm in diameter, the enhancing area is 59%, 42% and 27%, respectively (see Table 1).

As a result, the intensity distribution depends on the effective area, showing the characteristic strips of leaning nanowires and showing the uniformity and the regularity of the SERS signal. Thus, it can be concluded that for small-sized molecules, the main characteristic of the SERS substrates is a large percentage of the enhancing-substrate area and uniformity of SERS-spectrum amplification over the substrate area.

Figure 9b shows histograms of the area distribution of the phthalocyanine SERS peak at 1350 cm^−1^. In contrast to a solution of MG molecules, the dependence of the amplification of the SERS spectra of phthalocyanine on the NW diameter is nonmonotonic, with a maximum at NW diameter of about 100 nm. If we compare the values of the amplifying area for substrates with NWs of different diameters, the greatest number of high-intensity peaks appears on the substrates with NWs 100 nm in diameter. It should be noted that the size of phthalocyanine molecules (2.5 nm) is comparable to the size of the hot spot that appears in the gap between the leaning NW tips.

In this case, the main factor affecting the recording of a high-intensity SERS spectrum may be the angular dependence between the hot spot and the phthalocyanine molecule (at which angle the molecule was adsorbed relative to the hot spot region). Due to their high elasticity, NWs with diameters of 60 and 100 nm form agglomerates, consisting of a large number of leaning NWs. In such a bundle of leaning NWs, different angles between their tips may appear, which would also affect the nature of the hot-spot formation and subsequent amplification of the SERS spectra.

The fact that the main factor in the amplification of the SERS spectra of macromolecules exemplified by phthalocyanine is precisely the angular dependence rather than the area of the enhancing surface, is demonstrated in Figure 7. For phthalocyanine molecules with a concentration of 10 mM, there are distribution patterns of the area under three characteristic peaks on substrates with NWs of 60, 100 and 200 nm in diameter. Let us take a closer look at the SERS-mapping of the peak at 1350 cm^−1^. The enhancing areas are 63%, 77%, 61%, and the relative shifts of the mean value (*n*) are nearly 25%, 3%, 23% for substrates with NWs of 60, 100, and 200 nm in diameter, respectively (see Table 1). The values for NW substrates with diameters of 60 and 200 nm practically coincide, while for a NW with a diameter of 100 nm they differ significantly. In addition, for 100 nm diameter NWs, the value of the histogram mode differs by more than an order of magnitude from the corresponding values at 60 and 200 nm diameter NWs (Figure 9b). This confirms the hypothesis about the importance of the distribution of local electric fields near the leaning NW tips.

**Figure 10:**
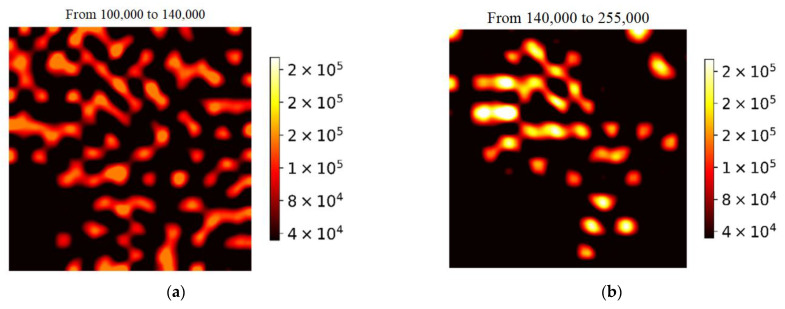
(**a**) Histograms of the distribution of the area value of the MG SERS peak located at 1177 cm^−1^. (**b**) Histograms of the distribution of the area value of the phthalocyanine SERS peak located at 1350 cm^−1^.

Let us consider in more detail the intensity-distribution histogram for MG molecules adsorbed on the SERS substrate with 60 nm diameter NWs. In addition to the first peak on the histogram, which characterizes the SERS signals close to the spectral noise, two enhancement ranges can be distinguished: 1.0^5^–1.4^5^ and 1.4^5^–2.5^5^. SERS-mapping images were plotted for these enhancement ranges, and there is a significant difference between them (Figure 10). Image analysis suggests that these areas correspond to different mechanisms of SERS amplification. The areas of the greatest enhancement correspond to hot spots of the gap type, which appear when the tips of two or more NWs lean toward each other at certain angles. They correspond to the horizontal stripes in the SERS-mapping image. The image shows that similar horizontal stripes appear when two adjacent NWs are leaning to each other by its tips (Figure 1c). The region of moderate enhancement corresponds to tip hot spots that appear at the NW tips. The conditions for their occurrence are simpler, and, therefore, the total area of such type of amplification region is larger than for hot spots of the gap type.

**Figure 11:**
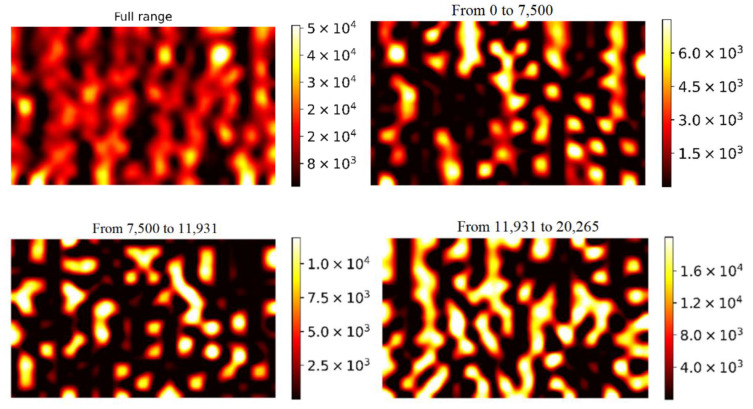

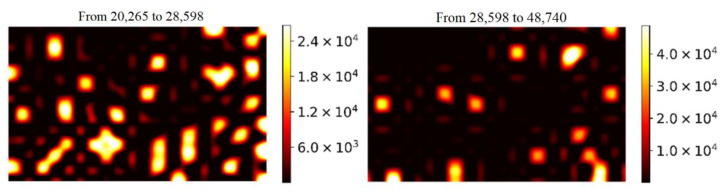
SERS-mapping of the three enhancement zones of 10 mM phthalocyanine molecules adsorbed on substrates with 100 nm diameter NWs.

In the case of phthalocyanine molecules, a similar analysis was carried out for a SERS substrate with 100 nm diameter NWs (Figure 11). In contrast to MG molecules, the size of a phthalocyanine molecule is in the order of, or larger than, the size of a hot spot region. The angles at which the molecule is adsorbed with respect to the hot spot region, as well as the angle at which neighbouring NWs lean together, become of fundamental importance for amplifying the SERS signal. Therefore, many peaks can be distinguished on the intensity-distribution histogram, apparently associated with different angles between neighbouring NWs, which form hot spots of the gap-type. Due to the wide variety of such angles, there are more than two intensifying zones.

Taking all the obtained results, we assume that the developed SERS-mapping approach is applicable for a broad range of nano-objects, including biorelevant/biomimetic molecules as well as nanoparticles (e.g., semiconductor quantum dots) [80,81].

## 4. Conclusions

This paper shows the potential of the SERS technique for the spectrochemical analysis of large organic molecules, by using SERS-mapping on silver-NW metasurfaces, prepared by template synthesis in nanopores of tracking polymer membranes. The method has been tested with relatively “small” (malachite green, MG) and “large” biorelevant/biomimetic (phthalocyanine bearing eight bulky 2,6-diisopropylphenoxy groups, H_2_Pc*) molecules.

It is shown how the size of the studied molecules affects the choice of parameters of the enhancing surface to obtain the most intense SERS spectra. When choosing an enhancing substrate, it is important to know not only the theoretical enhancement factor, but also the repeatability of the signal over the substrate surface (effective amplifying area).

The optimal nanowire diameter for obtaining the optimal substrate was found. For MG, the optimal NW diameter was 60 nm, since, due to the surface topology features, the number of hot spots were the highest. For H_2_Pc*, the optimal NW diameter was 100 nm, since the most important factor was the orientation of the molecules with respect to the contacts between leaning nanowires (angular dependences).

While the size of analyte molecules is relatively small (several angstroms) in comparison with the size of the hot spot between the tips of leaning NWs, one can estimate reproducible Raman spectra from different points of the SERS substrate, but with fluctuation of the enhancement coefficient. The situation becomes even more complicated when we deal with H_2_Pc* analyte molecules that are larger than hot spots in size. In this case, one can estimate SERS spectra which have different shapes depending on the orientation and spatial location relative to hot spots of SERS-surfaces. Reliable SERS-fingerprinting of analytes has been hindered by poor reproducibility and uniformity of substrates, which frequently leads to large variations of signal from different points of the substrate.

All these questions can be clarified by obtaining a full image- surface map of Raman spectra over the whole SERS substrate. In this case, it is possible both to give a quantitative characteristic of the enhancing SERS surface and find the robust spectrum of the analyte even at extremely low concentrations. For the standard organic dye MG, a stable amplification of all spectral peaks at different substrate points was demonstrated. At the same time, the SERS spectrum of H_2_Pc* macromolecules has the feature that different peaks of the spectrum are amplified at different points of the substrate, because the macromolecules are adsorbed at different angles to the leaning NWs, and various parts of the molecule fall into the region of the enhanced field. However, the large area mapping and subsequent averaging of the spectra make it possible to obtain the SERS spectrum close to the Raman spectrum of H_2_Pc* and thereby detect molecules with low concentrations.

It was found that, for molecules of different sizes, the efficiency of SERS amplification depends on various factors: the area of effective SERS amplification and the angular dependence between the tips of the leaning NWs. The first factor is crucial for small-sized molecules, while the second factor is essential for large-sized molecules. It has been shown that substrates with a small NW diameter (60 nm) are better suited for small-sized molecules of the MG-dye type since they provide the maximum area of effective SERS amplification. Thus, dye molecules can be adsorbed even in the smallest gaps between agglomerated NWs. For large molecules, H_2_Pc*-NWs with a diameter of 100 nm are better suited. They have the optimal size of the gap and the angle between their tips, thus providing the best amplification of the SERS signal for large-sized molecules.

## Figures and Tables

**Table 1:** Summary of the investigated SERS-active substrates efficiency.

Adsorbed Molecules	NW Diameter, nm	Noise Threshold	Enhancing Area, %	Mean	Median	*η*, %
MG	60	87,764	59	100,722	98,750	1.96
	100	5600	42	63,293	50,685	19.92
	200	3248	27	4884	2456	49.71
Phthalocyanine	60	3989	63	6900	5188	24.81
	100	6060	77	13,068	12,720	2.66
	200	963	61	1630	1251	23.25

## Data Availability

The data presented in this study are available on request from the corresponding author.

## Supplementary Materials

The following supporting information can be downloaded at: https://www.mdpi.com/article/10.3390/bios13010046/s1, Figure S1: Displacement vectors corresponding to selected vibrations in Raman spectrum of H2Pc* according to quantum-chemical calculations performed in gas phase at BP86/def2-SVP level. The normalized activities are given in brackets; Figure S2: Mapping at different peaks on SERS substrates; Figure S3: Mapping at spectral peak at 688 cm^–1^; Figure S4: (a) comparative spectra of phthalocyanine molecules, recording using a 532 and 1064 nm operating wavelength lasers as an excitation source. (b) comparative SERS and Raman spectra of phthalocyanine molecules. (c) spectra of phthalocyanine molecules, taken at 60, 100 and 200 nm diameter NWs, when exited by a 1064 nm operating wavelength laser. The references in Supplementary Materials were cited in Refs. [38,64,65,82,83,84,85].
