# Supplementary material for: Ultrasensitive Optical Fingerprinting of Biorelevant Molecules by Means of SERS-Mapping on Nanostructured Metasurfaces
Source: Biosensors (Basel). 2022 Dec 28;13(1):46. doi: 10.3390/bios13010046 (PMC9855407; doi:10.3390/bios13010046)
Supplement: Supplementary file 1 [file biosensors-13-00046-s001.zip › biosensors-2033912-supplementary.pdf]

Synthesis of Ag-NWs-Substrate

A feature of the template synthesis method is that the parameters of the obtained secondary nanostructures (NWs in our case) completely duplicate the parameters of the TM used as a template. It is also worth noting that in the manufacture of metasurfaces with NWs, due to capillary forces and effects after etching and drying, NWs lean together, forming agglomerates along the irradiation strips (due to the peculiarities of TM irradiation) [1–3].

Ag-NWs array on TM was manufactured in the following way. A thin silver coating of 50–70 nm was deposited on one side of the TM by thermal vacuum deposition to create a conductive layer. Then, a copper layer with a thickness of about 10 μm was electrochemically deposited on the silver-coated side of TM. The next step was the electrochemical deposition of silver using the commercial silvering electrolyte (KLIO Company, Russia) into the pores of the TM. The deposition was carried out in galvanostatic mode at a constant current value of 4 mA/cm^2.

In the process of metal deposition, the cell voltage was monitored by Elins P30S potentiostat (Chernogolovka, Russia), that allowed it to set and to control the length of the Ag-NWs during the growth process. The length of Ag-NWs of 100 and 200 nm diameter was chosen (to achieve the desired structure of hot spots) to be 10 μm, that is the thickness of the using TM, and so the biggest possible value of the Ag-NWs length. For the 60 nm diameter Ag-NWs its length was chosen to be 2.5 μm due to the features of its leaning process for the effective hot spots' formation.

At the end of the galvanic deposition process, the TM with an array of Ag-NWs inside its pores was washed from the electrolyte and then etched in a concentrated alkali solution (6M NaOH) at a temperature of 85° C. After the template etching, the metal substrates with the array of Ag-NWs were kept in 5% HCl to neutralize traces of alkali and then were washed in distilled water.

Molecules and Samples

Synthesis. Phthalocyanine H_2Pc^* was synthesized in 48% yield using template condensation of 4,5-bis(2,6-diisopropylphenoxy)phthalonitrile with magnesium turnings in refluxing absolute pentanol and subsequent demetallation of the resulting magnesium(II) complex with trifluoroacetic acid (see supporting information for more details). Solutions of this phthalocyanine with concentrations 10^{-4}, 10^{-6} and 10^{-8} M were prepared in chloroform, which was distilled over K_2CO_3 and filtered through alumina layer.

Computational details. Geometry optimization was performed using ORCA 5.0.0 package [4] with BP86 functional and def2-SVP basis set [5] with tight SCF criteria. Numerical frequency calculations of Raman spectrum of H_2Pc^* were made at the same level of theory to determine Raman wavenumbers and activities. See details in the next section.

Synthesis of octa(2,6-diisopropylphenoxy)phthalocyanine H_2Pc^*:

4,5-bis(diisopropylphenoxy)phthalonitrile (120 mg, 0.25 mmol) and magnesium chunks (12mg, 0.5 mmol) were suspended in 5 ml of dry pentanol, mixture was degassed and refluxed under argon atmosphere for one day. After cooling of reaction mixture to 85 °C, 0.5 ml of trifluoroacetic acid was added and mixture was heated stirred at this temperature for 10 min, until the single Q-band of the magnesium complex vanished at 680 nm in the UV-Vis spectrum of the reaction mixture sample and Q-bands of metal-free complex appeared at 701 and 665 nm. After cooling to room temperature, mixture was diluted with 20 ml of 50% aq. EtOH, the formed precipitate was filtered, washed with EtOH and chromatographed on silica using chloroform/hexane 1:1 vol. mixture. H_2Pc^* was isolated as a grass-green powder (58 mg, 48%). Its spectral characteristics agree with the previously reported data [6].

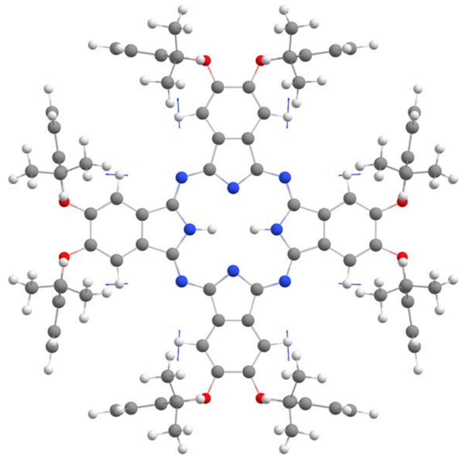

1070.72 cm^{-1} (0.07)

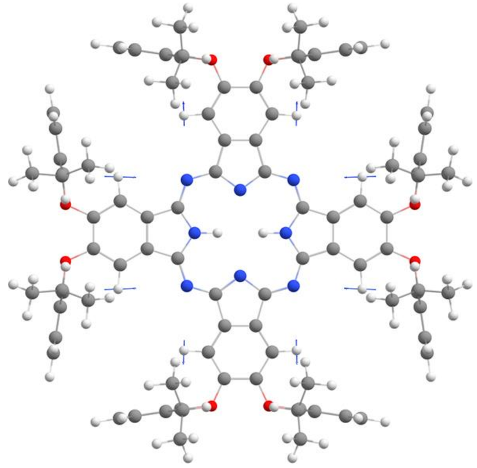

1099.05 cm^{-1} (0.10)

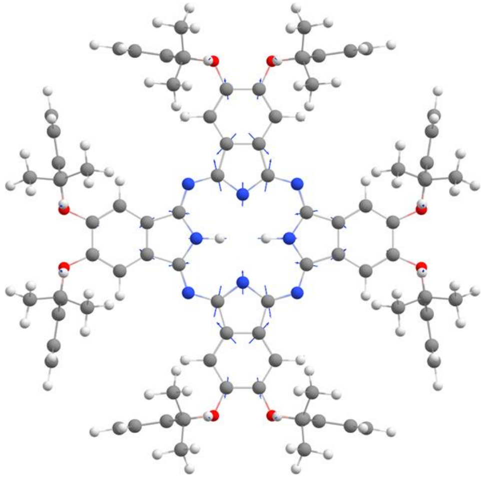

1325.77 cm^{-1} (1.00)

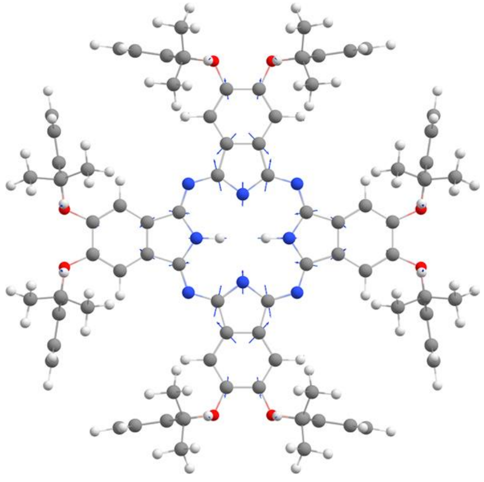

1367.79 cm^{-1} (0.42)

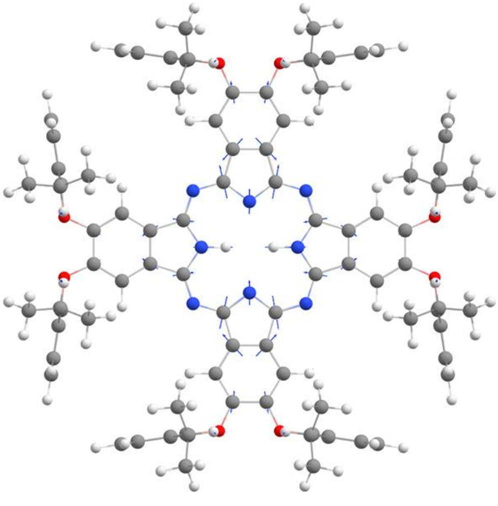

1464.46 cm^{-1} (0.18)

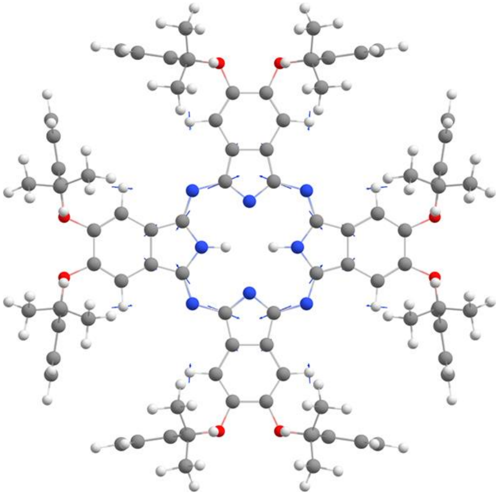

1522.66 cm^{-1} (0.18)

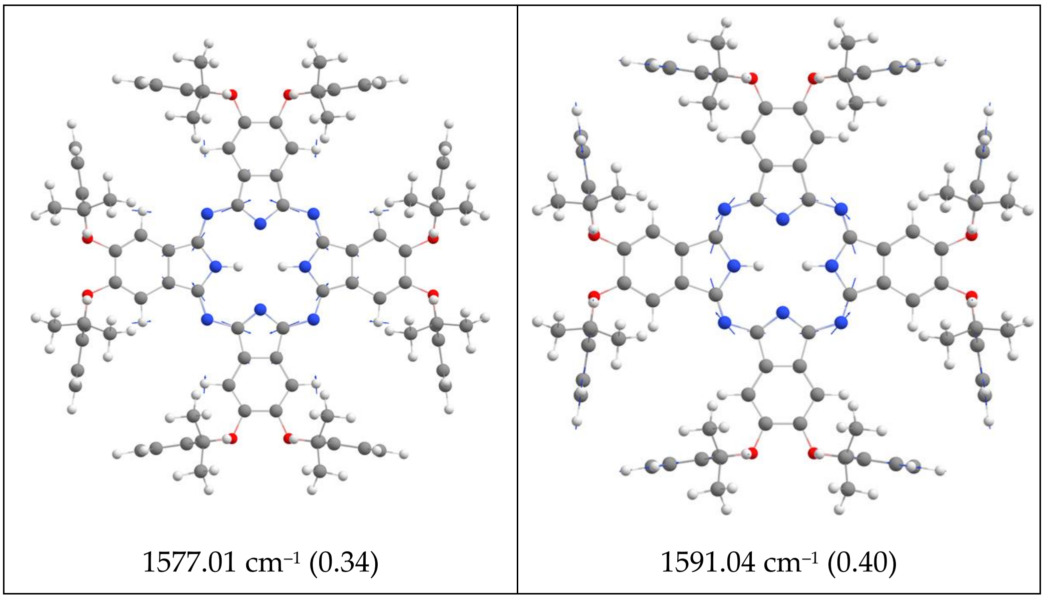

Figure S1. Displacement vectors corresponding to selected vibrations in Raman spectrum of H_2Pc^* according to quantum-chemical calculations performed in gas phase at BP86/def2-SVP level. The normalized activities are given in brackets.

SERS Mapping of MG Molecules Adsorbed on SERS Substrates

Here for MG molecules, it is well seen that for different bands SERS mapping on the same substrate (60 nm, for example) patterns are the same.

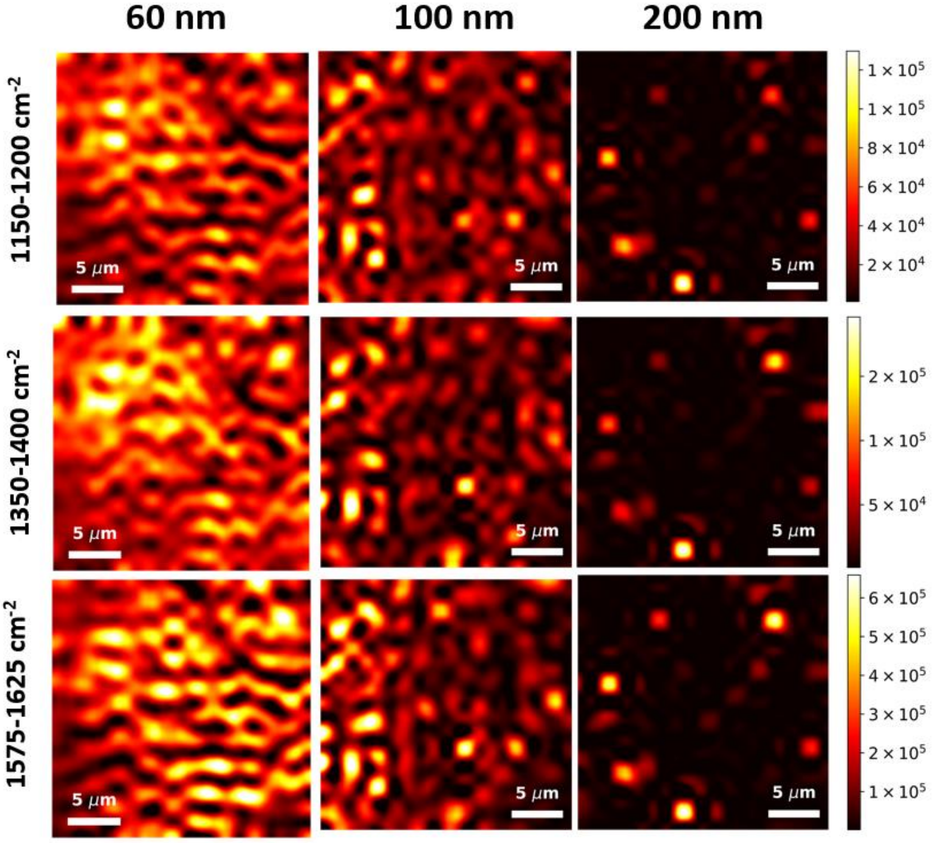

Figure S2. Mapping at different peaks on SERS substrates.

Raman Spectral Maps of the H_2Pc^* Solvent on SERS Substrates

Figure S3 shows the SERS maps plotted corresponding to the intensity of this 688 peak for H_2Pc^* (10^{-4} mol) adsorbed on SERS-substrates with NWs 60, 100, and 200 nm in diameter. On substrates with NWs 100 and 200 nm in diameter, the presence of this peak in the SERS mapping is most noticeable, which is also seen in the SERS spectra. H_2Pc^* is known to form unique cubic clathrate crystal packing with almost 40% of free space filled with solvent molecules [7], thus we can expect that films formed after deposition of 10^{-4} M solution onto SERS substrate contain such clathrates capturing chloroform molecules. On the other hand, formation of these crystalline particles near hot spots can be responsible for close resemblance of SERS Raman spectra with unenhanced spectra measured for a cast film.

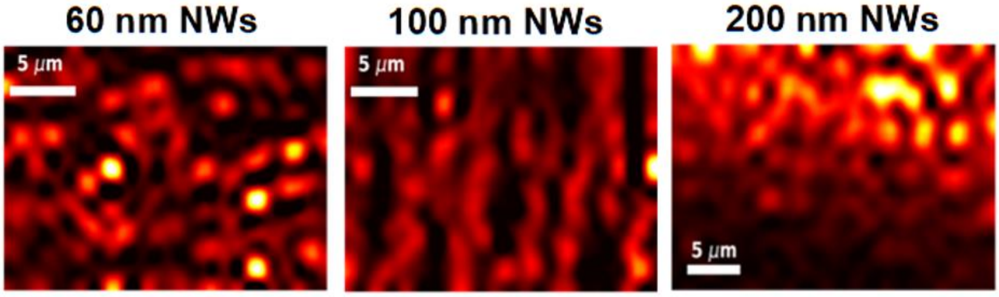

Figure S3. Mapping at spectral peak at 688 cm^{-1}.

Dependence on Excitation Wavelength

Figure S4a presents comparative spectra of phthalocyanine molecules on glass, recording using a 532 and 1064 nm operating wavelength lasers as an excitation source. This comparison was made to diagnose peaks resolution in the phthalocyanine molecules spectrum. Although phthalocyanine molecules have luminescence in the optical range, the peak resolution, exited by the laser operating at 532 nm, in spectrum is almost the same as by 1064 nm. This suggests that the substrates actively suppress luminescence. It has been shown that, by exited the spectrum in the infrared region, less noise is detected.

Among other things, it should be noted that the increase of intensity in SERS spectrum, compared to the Raman spectrum, is noticeable (exited by 1064 nm laser) (Figure S4b). Than Raman spectrum was obtained in the area of the substrate, where there are no NWs, but only a thin silver layer applied by thermal spray as one of the first steps of substrate' synthesis. When applying a solution with phthalocyanine molecules to the substrate, the droplet is spreading, and comparing the SERS spectrum with the Raman obtained on the glass is not correct. Therefore, there was a need to record the spectrum of phthalocyanine at the same substrate in the area, where there is no NWs, for objective comparison. This indicates the effective enhancement of local electrical fields in the infrared area by silver NWs with a diameter of 100 nm.

Figure S4c shows the spectra of phthalocyanine molecules, taken at 60, 100 and 200 nm diameter NWs, when exited by a 1064 nm operating wavelength laser.

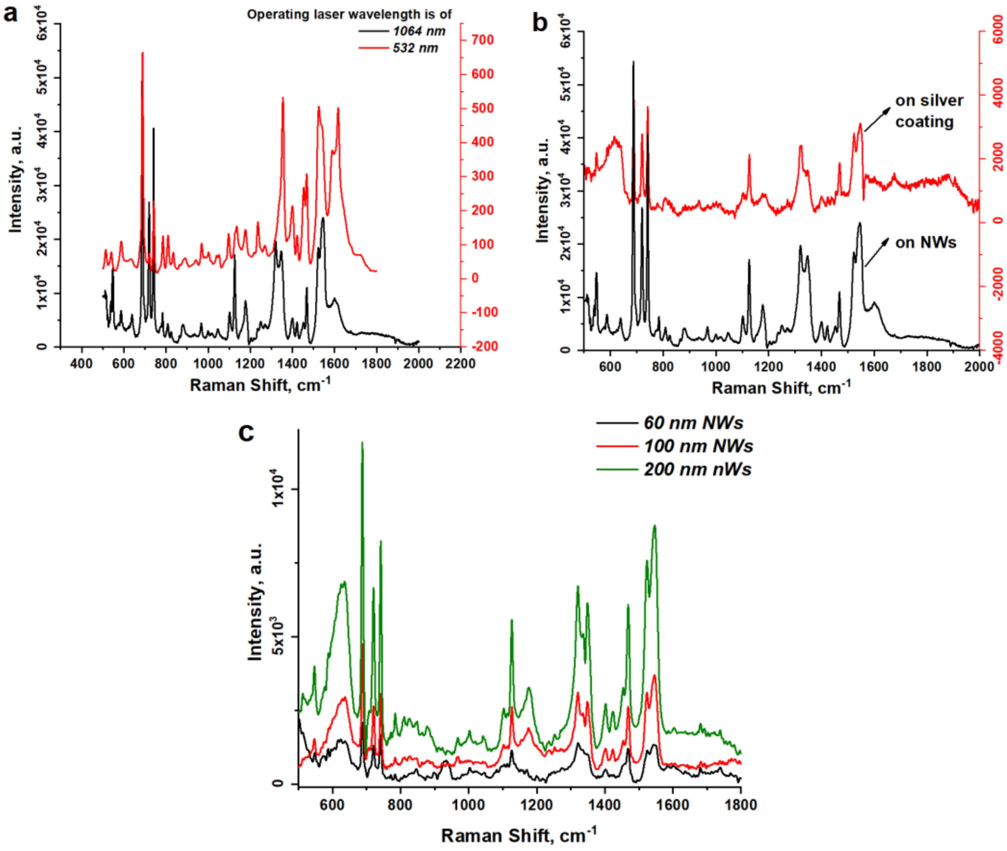

Figure S4. (**a**) comparative spectra of phthalocyanine molecules, recording using a 532 and 1064 nm operating wavelength lasers as an excitation source. (**b**) comparative SERS and Raman spectra of phthalocyanine molecules. (**c**) spectra of phthalocyanine molecules, taken at 60, 100 and 200 nm diameter NWs, when exited by a 1064 nm operating wavelength laser.

References

1. Razumovskaya, I.V.; Kovalets, N.P.; Bedin, S.A.; Grigor'ev, Y.V. Agglomeration of Nanowires on a Substrate for Surface-Enhanced Raman Scattering. *J. Exp. Theor. Phys.* **2021**, *132*, 818–823, doi:10.1134/S1063776121050058
2. Kovalec, N.P.; Kozhina, E.P.; Doludenko, I.M.; Razumovskaya, I.V.; Bedin, S.A.; Grigoriev, Y.V.; Kanevsky, V.M. Agglomeration of Ensembles of Silver Nanowires, Obtained by the Method of Template Synthesis. *Bull. Russ. Acad. Sci. Phys.* **2021**, *85*, 854–857, doi:10.3103/s1062873821080116.
3. Kozhina, E.P.; Bedin, S.A.; Nechaeva, N.L.; Podoynitsyn, S.N.; Tarakanov, V.P.; Andreev, S.N.; Grigoriev, Y.V.; Naumov, A.V. Ag-Nanowire Bundles with Gap Hot Spots Synthesized in Track-Etched Membranes as Effective SERS-Substrates. *Appl. Sci.* **2021**, *11*, 1375, doi:10.3390/app11041375.
4. Neese, F. Software update: the ORCA program system, version 4.0. *Wiley Interdiscip. Rev. Comput. Mol. Sci.* **2018**, *8*, doi:10.1002/wcms.1327.
5. Weigend, F.; Ahlrichs, R. Balanced basis sets of split valence, triple zeta valence and quadruple zeta valence quality for H to Rn: Design and assessment of accuracy. *Phys. Chem. Chem. Phys.* **2005**, *7*, 3297–3305, doi:10.1039/b508541a.
6. Martynov, A.G.; Mack, J.; Ngoy, B.P.; Nyokong, T.; Gorbunova, Y.G.; Tsivadze, A.Y. Electronic structure and NH-tautomerism of a novel metal-free phenanthroline-annelated phthalocyanine. *Dye. Pigment.* **2017**, *140*, 469–479, doi:10.1016/j.dyepig.2017.01.072.
7. McKeown, N.B.; Makhseed, S.; Msayib, K.J.; Ooi, L.L.; Helliwell, M.; Warren, J.E. A phthalocyanine clathrate of cubic symmetry containing interconnected solvent-filled voids of nanometer dimensions. *Angew. Chem. Int. Ed.* **2005**, *44*, 7546–7549, doi:10.1002/anie.200502668.
